# Time-variant Granger causality analysis for intuitive perception collision risk in driving scenario: an EEG study

**DOI:** 10.3389/fnins.2025.1604751

**Published:** 2025-06-19

**Authors:** Zhe Wang, Jialong Liang, Shang Shi, Peng Zhai, Lihua Zhang

**Affiliations:** ^1^Academy for Engineering and Technology, Fudan University, Shanghai, China; ^2^Department of Computer Science, University College London, London, United Kingdom; ^3^Engineering Research Center of AI and Robotics, Fudan University, Shanghai, China

**Keywords:** functional connectivity, intuitive prediction, collision risk, EEG, Granger causality

## Abstract

Intuition is a rapid and unconscious cognitive process that is widely utilized in driving scenario. The current study examines the neural mechanisms behind intuitive driving by performing a time-varying Granger causality analysis on source-domain EEG data. We construct an innovative experimental setup that utilizes immersive driving simulation videos to elicit intuitive decision-making alongside with neural activities. We performed Granger causality analysis on a sliding window basis that resulted in a directed connectivity model. By examining the node strength, we identify that the experienced drivers increase activation in intrinsic functional networks associated with visual attention and decision-making, which can be considered as the evidence for possessing better collision risk perception when compared to novice drivers. We also identify that experienced drivers exhibit a more stable and dispersed connectivity, especially in the beta band. In contrast, novice drivers exhibited more complex and less efficient connectivity, which can be interpreted as evidence of more efficient neural strategies for rapid decision-making in experienced drivers. This work not only advances the understanding of intuitive driving but also offers valuable insights for developing intelligent driving hazard perception systems. By targeting individual differences, we pave the way for personalized training programs to enhance driving safety and performance.

## 1 Introduction

In everyday life, we face numerous situations that require decisions, with many of the simpler choices being based on gut instinct, or “intuition" (Kahneman, 2011). Theoretically, intuition stands as a cornerstone of human cognition, intricately guiding our decision-making and problem-solving endeavors (Hosseinzadeh Lotfi et al., 2023). Intuitive decision-making is quick and effortless, but it can introduce biases that undermine the logical soundness and accuracy of the decisions (Newell and Shanks, 2014). The dual-process theory posits that human cognition operates via two distinct systems: System 1, characterized by rapid, intuitive, and automatic thinking, and System 2, which involves slower, analytical, and deliberate processing (Alter et al., 2007). Newell and Shanks (2014) propose a framework suggesting that decision-makers perceive the world through a “lens of cues," which mediates the relationship between environmental stimuli and internal perceptions. In 1977, Nisbett and Wilson's study indicates that individuals often lack introspective awareness of the cognitive processes underlying their intuitive judgments, suggesting a phenomenon where people are unaware of the existence of influential stimuli and the responses they elicit. Dane and Pratt describe intuition as judgments emerging from swift, non-conscious, and holistic associations (Dane and Pratt, 2007). Evans et al. (2010) characterize intuition as an immediate form of understanding that contrasts with analytical reasoning. In their study employing an investment game paradigm, they discovered that when participants' self-control resources are depleted, they tend to favor default options (Evans et al., 2011). Jia et al. investigate the influence of cognitive limitations and contextual induction on intuitive decision-making through a questionnaire-based experiment. Participants answered eight multidisciplinary single-choice questions while their behavioral patterns and EEG responses were recorded and analyzed. In the post-processing stage, EEG data were analyzed using machine learning techniques, specifically k-nearest neighbors (kNN), for offline classification (Jia et al., 2023). Results indicate that individuals exhibit different decision-making styles–rational, mixed, or intuitive–based on the context and their cognitive resources. However, the study does not fully consider individual differences that could influence decision-making patterns. While intuitive decision-making is efficient, it may sometimes clash with analytical reasoning, potentially giving rise to cognitive challenges such as selective attention bias, the endowment effect, and framing biases. However, when cognitive resources are limited and deliberate analysis is impractical, relying on intuitive processing can actually enable quicker and, in some cases, more precise evaluations.

Driving intuition is a fundamental example of human cognitive intuition in real-world scenarios and serves as a critical subject of study for understanding the mechanisms underlying the emergence and development of intuition in complex environments (Kuoch et al., 2018; Duma et al., 2017). Road traffic injuries represent a significant public health challenge and are among the leading causes of death and disability worldwide. In the meantime, according to the World Health Organization (WHO), these incidents claim over 1.3 million lives annually, equating to one fatality every 25 s, with a disproportionate impact in low- and middle-income countries (Rajčević et al., 2024; DeNicola et al., 2016). In light of these statistics, several studies have focused on driving intuition to better understand the brain mechanisms and potentially mitigate such tragedies. Chang et al. developed a multi-layer brain network (MLBN) model combined with oscillatory envelope-based functional connectivity metrics to analyze the dynamic process of driving, focusing on steering actions (left, right turns, and straight movements at intersections) while EEG signals were recorded. Results showed significant differences in MLBN structure and parameters among the steering conditions, suggesting the feasibility of using dynamic MLBN for driving behavior recognition (Chang et al., 2022), similar works are also in the detection of driver braking intention (Nguyen and Chung, 2019; Ju et al., 2022). Jiang et al. (2025) investigate the neural mechanisms of driver hazard recognition in rear-end collisions using EEG-based complex brain networks and proposes a two-stage threshold method for sparse representation of these networks to develop a deep learning model for hazard recognition state detection. Mora and Pino (2023) proposed a simplified prediction method for detecting emergency braking intentions using EEG signals and a convolutional neural networks (CNN) trained with a 2D matrices tensor arrangement. Hernandez et al. demonstrate the feasibility of using EEG signals to detect the intention to perform emergency braking under realistic driving conditions, where drivers experience cognitive states such as stress, workload, and fatigue. They achieve classification through support vector machine (SVM) and CNN, with a prediction accuracy of 71.8% (Hernández et al., 2018). Li et al. (2022) investigate drivers' EEG responses during emergency collision avoidance through a driving simulator experiment, analyzing EEG data across four stages of collision avoidance and finding significant changes in EEG activity, including gender differences in delta and alpha power ratios that indicate higher mental arousal in female drivers. The study's limitation of using a single collision event to avoid participant speculation and learning effects highlights the need for analyzing multiple emergency events to identify common brain patterns in collision avoidance. The rapid decision-making in response to danger encountered during driving, referred to as physical system intuition, differs significantly between experienced drivers and novices. Therefore, the intuitive neurophysiological differences between experienced drivers and novices represent an important area of research. Especially when exposed to immersive first-person perspective driving videos that simulate potential risks, the rapid collaborative decision-making and dynamic connectivity across various brain regions induced in these individuals provide valuable insights into the neural mechanisms underlying intuitive processes, which is of significant importance for subsequent studies.

In cognitive neurosciences, functional connectivity of brain network is conventionally estimated by classical methods, such as coherence, correlation, and synchronization metrics, based on temporal or frequency analysis over the EEG node space (Bowyer, 2016; Chiarion et al., 2023). Coherence, correlation, and synchronization metrics measure the similarity of oscillatory activity between brain regions, reflecting synchronized neuronal activity and functional interactions. However, these metrics do not reveal the directionality between neuronal firing and reception (Gao et al., 2015). Some techniques have been capable of estimating time-varying connectivity patterns without accounting for observational noise and non-stationarity (Gao et al., 2015). A comprehensive, data-driven modeling framework integrates state-space modeling with autoregressive models featuring time-varying coefficients, facilitating the estimation of time-resolved, renormalized partial directed coherence (PDC) in the frequency domain. This methodology quantifies both the direction and magnitude of dynamic network connectivity without presupposing the nonlinearity, non-stationarity, or stochasticity inherent in brain signals. However, identifying causal relationships among neural processes is often challenged by time-varying dynamics and observational noise. By applying Granger causality to phase shift events, researchers can achieve high temporal resolution in measuring directed connectivity, effectively distinguishing between periods of synchrony and desynchrony in neural activity (Baccalá and Sameshima, 2001; Marshall et al., 2014). The general concept of causality can be expressed in terms of expectability, with Granger causality typically computed by fitting a multivariate autoregressive (MVAR) model (Ding et al., 2000; Liang et al., 2000). Although MVAR model can be used to provide detailed characterization of dynamic relationships, it still has drawbacks, such as high model complexity, difficulty in parameter estimation, limitations of model assumptions, and computational cost. Additionally, model order selection is crucial in the analysis and is often determined using criteria such as the Akaike Information Criterion (AIC) and the Bayesian Information Criterion (BIC). These criteria offer several advantages, including balancing fit and complexity, preventing overfitting, and providing clear numerical indicators for easy comparison (Hesse et al., 2003). Among them, AIC tends to be more robust in situations with relatively small sample sizes when there are relatively few time series observations (Brewer et al., 2016). Moreover, AIC enhances the reliability and interpretability of Granger causality results by selecting the most concise model that sufficiently captures the temporal dependencies in the data, thereby balancing model fit with complexity to avoid overfitting. An alternative to using AIC for model order selection is to employ model order itself as a metric for Granger causality. Specifically, we define the causality relationship between maximum lag order and AIC as the difference in minimum description length between the restricted (only the past can influence the future) and unrestricted model (Hu et al., 2021). Previous research has shown that this approach (unified Granger causality analysis, uGCA) has been successfully applied to dynamic brain networks, and appear to have better stability than the traditional approach (Li et al., 2023). We chose to select the traditional Granger causality approach mainly for its ecological validity, but uGCA is a highly suitable metric alternative in follow-up research. In addition, it quantifies the time-dependent network connectivity without relying on any a priori assumptions about the nonlinear, non-stationary, and stochastic nature of brain signals. The efficacy of the AIC-based Granger causality approach has been corroborated in the study.

The main contributions of the current study are as follows:

- Naturalistic control of intuition use: the current study innovatively utilizes an objective measure (the possession of a driving license) to control for the level of real-life driving experience.
- Innovative design of immersive driving stimuli: the current study utilized realistic first-person perspective driving videos that require rapid decision making. This mimics real-world driving conditions well.
- Directed graph network to track causal effects: the study innovatively utilized Granger causality analysis in a dynamic network. This allows us to examine how the functional network organization changes across the decision making process.

## 2 Materials and methods

In this section, we describe the experimental procedure and data analysis procedure of the current study.

### 2.1 Subjects

The current study recruited 23 right-handed volunteers (23.57 ± 2.39 years old). Among them, 12 (seven males and fiv females) held a valid driver's license, while the remaining 11 participants (six males and five females) do not. We considered gender during the participant recruitment phase to ensure that each group includes both males and females, thereby minimizing the potential confounding effects of gender. There were 40 trials in the experiment. Each trial began with a 3-s black screen as a preparation phase, followed by the video presentation phase. Participants were instructed to watch the video and anticipate potential car collisions. They were instructed to press the spacebar when they consider a collision to be inevitable, and the reaction time were recorded. When perceiving a collision risk event, volunteers were asked to press the spacebar to record the reaction time, with their hand resting on the spacebar to minimize movement-induced interference. Additionally, participants are informed that each video randomly contains a hazardous event with a 50% probability. To reduce fatigue effects, a 5-min break is provided at the midpoint of the experiment. All participants provided written informed consent before the study commenced, which detailed the experimental procedures and study duration to ensure full understanding and voluntary participation. This study was approved by the Ethics Committee of Fudan University for biomedical research projects (Approval No. FE241791).

### 2.2 Experiment design

The video stimuli that used to induce participants' intuitive driving predictions were crucial to the success of research on video-induced driving intuition. In this study, we employed the non-commercial BeamNG.drive vehicle simulation tool to generate a series of video clips, which were then presented to participants to elicit intuitive driving responses. Specifically, 47 car collision and 49 non-crash original video clips were initially recorded from a first-person driving perspective using screen recording. Considering that clips featured low driving speeds and a more cautious driving style, a subset of the footage was excluded. Ultimately, we retained 20 clips depicting accident scenarios and 20 clips illustrating smooth, incident-free driving clips until completion, resulting in a total of 40 video clips. Finally, these video clips (trials) were presented in a randomized order to reduce expectation bias, enhance result applicability and balance emotional responses.

In this experiment, the experimental procedure was developed using the MATLAB-based Psychtoolbox (Misirlisoy, 2016). MATLAB, a powerful programming language, offers flexible data processing and precise timing control capabilities. Psychtoolbox, specifically designed for psychological and behavioral experiments, provided efficient functions for visual stimulus presentation, response collection, and experimental workflow management. By integrating MATLAB with Psychtoolbox, we achieved precise control over the playback, pause timing, and decision intervals of each video segment, ensuring consistency across all experimental phases. When assessing participants' intuitive car collision risk abilities, Psychtoolbox facilitated accurate pause control and decision duration settings for the videos, maintaining high experimental precision.

**Figure 1:**
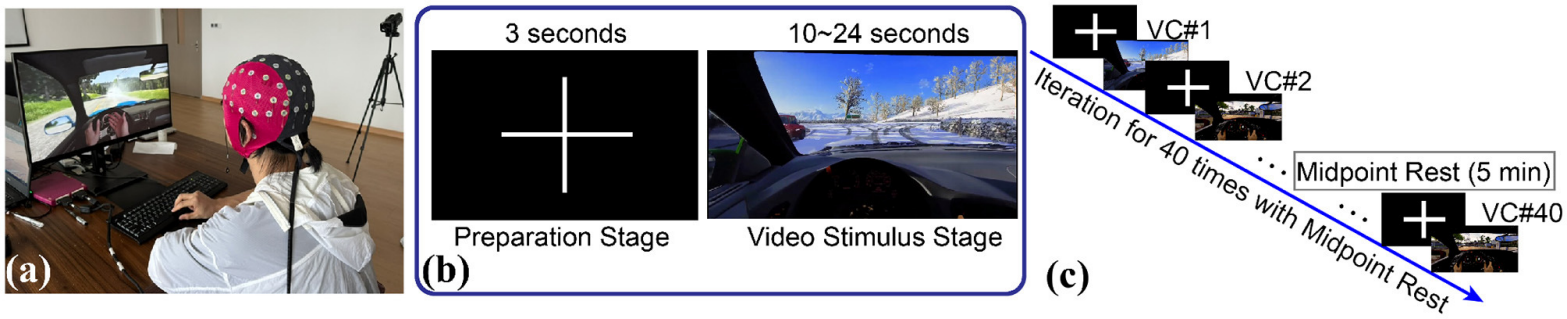
Experimental process of the car collision-induced car collision experiment: **(a)** Car collision prediction scenario; **(b)** Two repeated experimental stages; **(c)** Schematic diagram of the experimental procedure with EEG recording.

Regarding equipment, this study utilized an Alienware 15R4 laptop with 32 GB of RAM and an NVIDIA GeForce GTX 1070 graphics card. An external 27-inch 4K 60Hz monitor was used to display experimental content to participants. The 4K resolution ensured clear video imagery, allowing participants to observe minute details, especially when determining the direction of a ball's trajectory. The laptop's built-in display served for operational control, presenting the experimental progress and control interface to ensure smooth execution. Combining high-performance hardware with precise software control effectively ensured the quality and accuracy of the experiment. The experimental process of the car collision induced driving intuition is illustrated in Figure 1.

### 2.3 Data pre-processing

In this study, EEG data are acquired using a standard 10–10 system cap equipped with 64 Ag-AgCl electrodes. The average potential across all electrodes served as the reference signal. Data collection is performed with the ASA-lab EEG system (ANT Neuro, Enschede, Netherlands), applying a amplifier gain of 20 × and appropriate filtering. EEG signals are digitized at a sampling rate of 1 kHz and recorded for offline analysis. Prior to each experiment, electrode impedances are checked and adjusted as necessary to maintain values below 10 kΩ, ensuring optimal signal quality. In addition to EEG recording, the timestamps of keypresses made by participants during decision-making tasks are logged, starting from the initiation of video playback. This behavioral data facilitate the assessment of decision accuracy and reaction times, enabling further analysis of the relationship between neural activity and decision performance.

During data pre-processing, the raw EEG signals are first down-sampled to 500 Hz to reduce data volume and enhance processing efficiency. A bandpass filter is then applied to extract alpha and beta frequency bands by removing irrelevant frequency components. Epochs were segmented around critical events, defined as the midpoints of videos showing collisions or non-collisions. Each epoch captured the period from 2 s before to 1 s after the event. Only trials with correct predictions are included in the following graph network analysis, and trials with errors are excluded to ensure data integrity, as errors could introduce noise affecting subsequent analyses. Independent Component Analysis (ICA) is employed to identify and remove artifacts from ocular and muscular sources, and ICA decomposition is performed using the Infomax algorithm, which seeks to maximize the statistical independence of source signals, effectively separating mixed EEG data into independent components. Artifact components are identified based on their temporal and spatial characteristics and subsequently removed. The ICA process is optimized using the Faster and Adjust plugins to enhance artifact correction. Following ICA, interpolation and re-referencing procedures are applied to ensure consistent reference across all channels. Specifically, the Adjust plugin automatically classifies these components based on predefined spatial and temporal criteria. The Faster plugin statistically evaluates components using *z*-scores of features such as kurtosis, power spectrum deviation, and correlation with EOG channels, with a default rejection threshold of |*z*|>3. Components flagged by either plugin were reviewed and removed prior to further analysis. After pre-processing, data from two participants in the no-driving-license group are entirely excluded due to excessive artifacts or technical issues, resulting in a final dataset suitable for analysis. It should be noted that the two excluded participants were female. Although the exclusion of these two female participants from the novice group may raise potential concerns, we address these in two ways. On the one hand, this study does not involve any gender-related comparisons. On the other hand, the EEG data from these two female participants were of poor quality due to excessive movement and frequent blinking, as confirmed during a review of their EEG recordings.

### 2.4 Dynamic functional network construction

In this subsection, we detail the theoretical framework employed to construct and analyze dynamic brain networks. Here, we outline the process of constructing and analyzing dynamic brain networks. This procedure typically involves estimating node and graph metrics, as well as performing time-window sliced graph network analysis to examine connectivity patterns. The dynamic brain network includes both directed and undirected metrics, such as node strength, clustering coefficient, and betweenness centrality for undirected networks, as well as transfer entropy and Granger causality for directed networks. In this study, we primarily focus on the directed metric, specifically Granger causality, which enables the identification of directional influences between brain regions over collision prediction time.

Intrinsic networks, also known as intrinsic connectivity networks, refer to large-scale brain networks that maintain functional organization even in the absence of external tasks or stimuli. The Default Mode Network (DMN) is a well-known example of an intrinsic network, but other networks such as the Dorsal Attention Network (DAN), Ventral Attention Network (VAN), Frontoparietal Control Network (FCN), Limbic Network, Somatomotor Network, and Visual Network also exhibit intrinsic connectivity patterns. FCN also show task-dependent activity, meaning they can be recruited dynamically based on cognitive demands. Thus, while these networks are often part of the intrinsic system, their engagement can be both spontaneous (intrinsic) and task-evoked (extrinsic). In this study, we specifically focus on the car collision task-evoked brain region activation.

### 2.5 Directed functional connectivity analysis

In this subsection, we describe the techniques for computing directed network metrics, which allow for tracing the temporal evolution of brain connectivity during car collision trials.

Building on the principles of directed network analysis, this study leverages Granger causality to investigate directional influences in continuous EEG data for enhanced car collision prediction. Granger causality is a statistical method that evaluates whether past values of one time series can improve the forecasting accuracy of another time series, thereby indicating a directional influence from the former to the latter (Granger, 1969). It serves as a fundamental technique for identifying predictive relationships between temporal signals. Here, we assume the presence of two stationary EEG time series, *X*_*t*_ and *Y*_*t*_, within a multi-dimensional signal. To illustrate how incorporating the historical data of one signal can enhance the prediction of another, we employ both univariate and bivariate autoregressive (AR) models on the data. In the context of univariate AR modeling, we have


(1) $$\begin{array}{l} X_{t}=\overset{p}{\underset{i=1}{\sum }}\alpha_{1i}X_{t-i}+\varepsilon_{1t} \end{array}$$

(2) $$\begin{array}{l} Y_{t}=\overset{p}{\underset{i=1}{\sum }}\alpha_{2i}Y_{t-i}+\varepsilon_{2t} \end{array}$$

where the prediction errors ε_1*t*_, ε_2*t*_ depend only on the past of the own signal *X*_*t*_, *Y*_*t*_. In contrast to univariate AR modeling, bivariate AR modeling explicitly incorporates the lagged values of both *X*_*t*_ and *Y*_*t*_, as shown


(3) $$\begin{array}{l} X_{t}=\overset{p}{\underset{i=1}{\sum }}\alpha_{i}X_{t-i}+\overset{p}{\underset{j=1}{\sum }}\beta_{j}Y_{t-j}+\epsilon_{1t} \end{array}$$

(4) $$\begin{array}{l} Y_{t}=\overset{p}{\underset{i=1}{\sum }}\alpha_{i}Y_{t-i}+\overset{p}{\underset{j=1}{\sum }}\beta_{j}X_{t-j}+\epsilon_{2t} \end{array}$$

where *p* denotes the model order, α_*i*_ and β_*j*_ represent the coefficients, and ϵ_1*t*_, ϵ_2*t*_ indicate the residuals of the bivariate models. If incorporating the past values of *Y*_*t*_ results in a significant reduction in the prediction error (i.e., when the variance of ϵ_1*t*_ is notably lower than that of ϵ_2*t*_), it is concluded that *Y*_*t*_ Granger-causes *X*_*t*_. In other words, the prediction of a signal is generally based on its own historical data as well as the past values of the other signal. In both instances, prediction accuracy is quantified by the variance of the one-dimensional prediction errors when modeling $\Sigma_{X_{t}|X_{t}^{-},Y_{t}^{-}}$ and $\Sigma_{Y_{t}|Y_{t}^{-},X_{t}^{-}}$, which can be given by:


(5) $$\begin{array}{l} \Sigma_{X_{t}|X_{t}^{-},Y_{t}^{-}}=\text{var}\left(\epsilon_{1t}\right) \end{array}$$

(6) $$\begin{array}{l} \Sigma_{Y_{t}|Y_{t}^{-},X_{t}^{-}}=\text{var}\left(\epsilon_{2t}\right) \end{array}$$

If signal *Y*_*t*_ influences signal *X*_*t*_, the variance of the prediction error reduces when a two-dimensional model is used, incorporating the past values of *Y*_*t*_ to predict *X*_*t*_. The Granger causality from *Y*_*t*_ to *X*_*t*_, representing the linear dependence between the two signals, which can be defined as:


(7) $$\begin{array}{l} F_{Y_{t}\to X_{t}}=\ln\;\left(\frac{\Sigma_{X_{t}|X_{t}^{-}}}{\Sigma_{X_{t}|X_{t}^{-},Y_{t}^{-}}}\right)=\ln\;\left(\frac{\text{var}\left(\epsilon_{1t}\right)}{\text{var}\left(\epsilon_{2t}\right)}\right) \end{array}$$

when applying Granger causality using a temporal sliding window approach to EEG data, each analyzed individually to capture dynamic changes in directional connectivity over time. The maximum value between the two terms, *F*_*XY*_ = max(*F*_*Y*→*X*_, *F*_*X*→*Y*_), serves as a straightforward measure of the strength of directional and/or bidirectional interactions. Specifically, the evaluation process leverages vector autoregressive (VAR) models and integrates a range of methodological approaches, including the Yule-Walker equations, information criterion methods (e.g., AIC, BIC), structured VAR (SVAR), and machine learning-based techniques. In this work, AIC is utilized to determine the model parameters, which can be described as: In this work, we used AIC to determine the max-lag value used in the model, which can be defined as:


(8) $$\begin{array}{l} \text{AIC}=n\cdot \ln\;\left(\frac{\text{RSS}}{n}\right)+2k \end{array}$$

where *n* is the number of observations, RSS is the residual sum of squares, *k* is the parameter number. We calculated every AIC value over each channel pair, trial and time sliding window. The final reported AIC values are averaged across trials and sliding windows. For more details, please see the result Section 3.1.

In Granger causality analysis, selecting the optimal lag order *p*_0_ (i.e., max_lag) for mean AIC is critical. For each epoch across the two groups, Equation 8 iterates through a range of lag orders, computing AIC for each. The AIC balances model fit and complexity, and the lag order that minimizes the AIC is selected as *p*_0_. This approach effectively captures the causal relationships within the EEG data while minimizing the risk of overfitting. The trend of AIC values across lag orders is visualized graphically, highlighting the optimal lag order. In summary, the strategy for selecting the numerical value of *p*_0_ is outlined as follows:

1. For each experimental condition (e.g., experienced drivers vs. novices), the first five trials of car collision risk events are selected.
2. Within each trial, AIC values are calculated for every pair of frequency bands (i.e., α, β, γ), resulting in an *N*×*N* matrix after performing source localization of brain regions.
3. The AIC values are then averaged across the collected trials.
4. This process is repeated for different values of *p*, and *p*_0_ corresponds to the lowest average AIC is selected.

### 2.6 Granger causality over sliding windows

In this work, a sliding window approach is employed to analyze the temporal fluctuations in EEG signals, thereby uncovering the dynamic processes that underlie the integration and reorganization of the brain's functional networks. Specifically, car collision events occur randomly, with an overall incidence rate of 50% across all video clips. The continuous EEG data is segmented into trial-segmented epochs. Since Granger causality identifies predictive relationships over time, applying it to segmented time windows allows for the analysis of how causal interactions over brain regions change over all car collision risk stages. For each trial, Granger causality analysis yields directional measures that differentiate between inbound and outbound causal influences. In this study, EEG trials corresponding to actual car collisions are segmented around the baseline time point, *t*_*c*_, and subsequently extended in both temporal directions: the preceding time period (*t*_*c*_−*t*_ahead_) and the following time period (*t*_*c*_+*t*_after_). This results in a time window expressed as *t*_*epoch*_∈[*t*_*c*_−*t*_ahead_, *t*_*c*_+*t*_after_]. Temporal sliding windows with a time interval of *t*_wdw_ are applied to capture dynamic shifts in brain activity during the car collision risk event. Moreover, these sliding windows are applied with overlap, allowing for continuous tracking of the brain region interactions and ensuring a high temporal resolution across the entire event duration. This overlapping approach enables the detection of transient causal influences and captures rapid changes in brain connectivity during car collision event.

### 2.7 Directed connectivity network quantification

In brain connectivity analysis, various metrics such as eigenvector centrality, betweenness centrality, closeness centrality, and clustering coefficient are employed to assess different aspects of a node's influence and position within the network. In this section, we focus on the application of the network metric known as node strength (NS) within EEG brain network research utilizing Granger causality analysis. NS quantifies the cumulative weight of all connections that a specific node has with every other node, effectively measuring its overall connectivity within the dynamic brain network. By computing the sum of the weights of both incoming and outgoing connections, NS offers insights into the prominence and role of a node in information transmission. Mathematically, the node strength (*S*_*i*_) of node *i*, which can be classified by in-strength $S_{i}^{in}$ and out-strength $S_{i}^{out}$, can be symmetrically given by:


(9) $$\begin{array}{l} S_{i}^{in}=\overset{N}{\underset{j=1}{\sum }}F_{j\to i} \end{array}$$

(10) $$\begin{array}{l} S_{i}^{out}=\overset{N}{\underset{j=1}{\sum }}F_{i\to j} \end{array}$$

where *S*_*i*_ represents the node strength of channel *i*, *F*_*ij*_ is the Granger causality weight between node *i* and *j*, and the summation extends over all nodes *j* connected to node *i*. In directed networks, a node's in-degree represents the number of edges directed toward it, while the out-degree denotes the number of edges originating from it. Regarding the practice of representing NS by summing its in-strength and out-strength, i.e., $S_{i}=S_{i}^{in}+S_{i}^{out}$, this approach offers a comprehensive measure of the node's overall NS. During visualization, we selected only the edges with the top 5% highest connection strength in each plot (each band, collision condition, participant condition, and time window).

To comprehensively assess the diversity of network topologies, we employed several key metrics: the node clustering coefficient, which quantifies the degree of local interconnectedness among a node's neighbors; global efficiency, measuring the overall efficiency of information transfer across the entire network; and characteristic path length, indicating the average shortest path between all pairs of nodes, thereby reflecting the network's integration level. Firstly, the node clustering coefficient, which quantifies the degree to which a node's neighbors are interconnected, is defined as follows:


(11) $$\begin{array}{l} C_{i}^{w}=\frac{\sum_{j,k}w_{ij}w_{ik}w_{jk}}{\sum_{j,k}w_{ij}w_{ik}} \end{array}$$

where *w*_*ij*_ is the weight of the edge between node *i* and *j*.

Secondly, the global efficiency metric, which quantifies the overall efficiency of information exchange across a network, is defined as follows:


(12) $$\begin{array}{l} E_{global}=\frac{1}{n\left(n-1\right)}\underset{i\neq j}{\sum }\frac{1}{d_{ij}} \end{array}$$

Thirdly, the characteristic path length, which quantifies the average shortest distance between all pairs of nodes in a network, is defined as follows:


(13) $$\begin{array}{l} L=\frac{1}{n\left(n-1\right)}\underset{i\neq j}{\sum }d_{ij} \end{array}$$

where *n* is the number of nodes in the network, *d*_*ij*_ is the shortest weighted path between node *i* and *j*, and path length is defined as the inverse of cumulative weight.

### 2.8 Statistical methods

#### 2.8.1 *t*-tests for global network metrics

As for *t*-tests for global network metrics, specifically for global network metrics (global efficiency, modularity, and characteristic path length), we employ Welch's *t*-tests to compare experienced drivers with novices across conditions and time windows. This approach accommodates potential unequal variances between groups and provides robust statistical inference for whole-network properties. We compare mean value between licensed and unlicensed participants for each sliding window separately to capture temporal dynamics in network integration and segregation processes.

#### 2.8.2 Mixed-effects ANOVA for node-level metrics

For node-level metrics (in-strength, out-strength, and clustering coefficient), we implement a mixed-effects analysis of variance (ANOVA) model with driving experience (license status) as a between-subjects factor and brain region as a within-subjects factor. This approach offers substantially enhanced sensitivity compared to the alternative method of averaging node-level metrics across regions followed by *t*-tests, which would obscure regional-specific effects and reduce statistical power. The mixed-effects model can be formulated as:


(14) $$\begin{array}{l} Y_{ijk}=\mu +\alpha_{i}+\beta_{j}+{\left(\alpha \beta \right)}_{ij}+\epsilon_{ijk} \end{array}$$

where *Y*_*ijk*_ represents the network metric value for subject *k* with experience level *i* in brain region *j*, μ is the grand mean, α_*i*_ is the main effect of driving experience, β_*j*_ is the main effect of brain region, (αβ)_*ij*_ is their interaction, and ϵ_*ijk*_ is the error term. Our primary focus was on the main effect of driving experience (α_*i*_), as this directly addresses our hypothesis regarding qualitative differences in neural processing between experienced drivers and novices.

#### 2.8.3 Node-level *t*-tests

Following significant ANOVA results indicating main effects of driving experience on node-level metrics, we conduct follow-up *t*-tests for individual nodes to identify which specific brain regions contributed to the observed group differences. Due to the high dimensionality of these results (multiple metrics × multiple regions × multiple time windows), direct interpretation of all node-level statistics would be unwieldy. Therefore, we aggregate significant findings by functional network affiliation (Visual Network, DMN, VAN, FCN) and time window to identify patterns of group differences across neural systems and temporal dynamics.

This approach allowed for the systematic mapping of group differences onto established functional brain networks, facilitating interpretation within existing neurocognitive frameworks of attention, visual processing, and cognitive control.

#### 2.8.4 Multiple comparison correction

To control the family-wise error rate across multiple statistical tests, we implement False Discovery Rate (FDR) correction with a threshold of α = 0.05. The FDR procedure ranks all *p*-values in ascending order and determines significance thresholds that control the expected proportion of false positives among all rejected null hypotheses, providing an optimal balance between Type I error control and statistical power. For visualization purposes, significant effects are denoted with asterisks where single (*), double (**), and triple (***) asterisks indicate FDR-corrected *p*-values less than 0.05, 0.01, and 0.001, respectively.

## 3 Results

### 3.1 Granger causality model order

**Figure 2:**
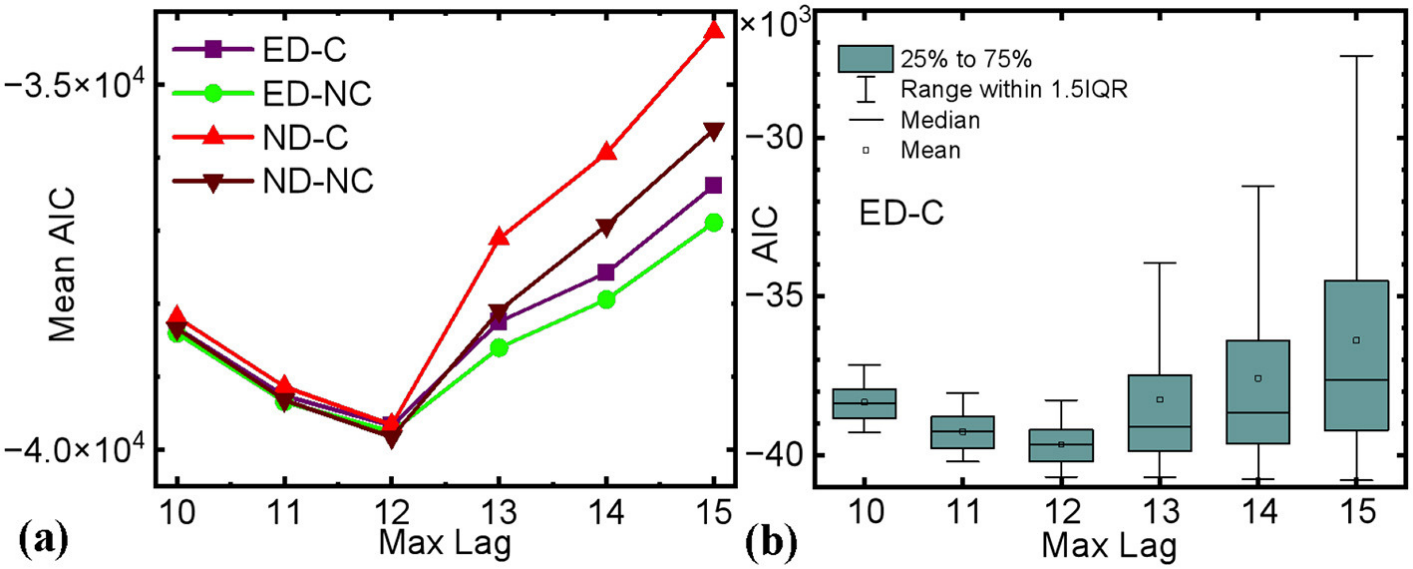
**(a)** The relationship between maximum sample lag and the mean AIC after traversing and averaging all 64-channel EEG channel pairs (ED-C, experienced drivers with collisions; ED-NC, experienced drivers with no collision; ND-C, novice drivers with collisions; ND-NC, novice drivers with no collision); **(b)** Boxplot showing the distribution of AIC values for different maximum lags.

Figure 2 presents a representative example of AIC-based model order selection for determining *p*_0_. Specifically, for each subject across all EEG electrodes, the optimal Granger causality model order is identified by minimizing the AIC within the range 1 ≤ *p* ≤ 15. The results indicate that the AIC consistently yielded reliable model order estimates across all trials. In this study, based on the trend observed in Figure 2, the optimal lag order is determined to be *p*_0_ = 12.

Figure 2a illustrates the results of the AIC-based model order selection procedure for determining the optimal maximum lag for Granger causality across all conditions. It should be emphasized that, after EEG source localization, AIC values were computed over the maximum lag value to strike a balance between model performance and computational complexity across all electrode pairs. Initially, we tested maximum lag values ranging from 1 to 15 using a small subset of trials. Subsequently, we refined the range to 10 ≤ *p* ≤ 15 to identify a precise local optimum. Ultimately, the selected AIC values stemmed from a random selection of 10% of all sampled trials, thereby reducing computation time while traversing all electrode pairs.

### 3.2 Granger causality-derived node strength in directed connectivity networks

**Figure 3:**
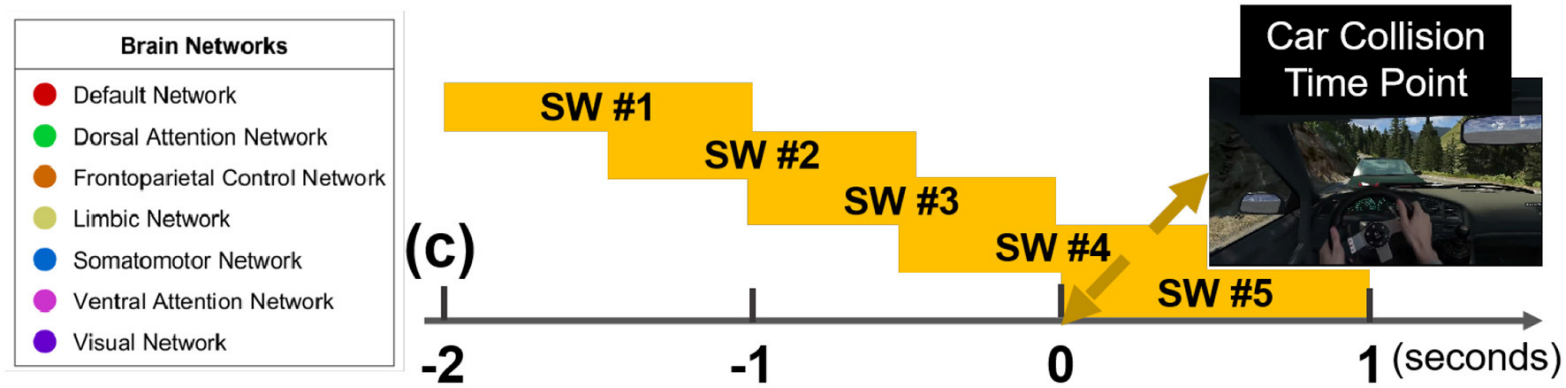
Granger causality based node strength networks during car collision trials in alpha band with five sliding windows, aligned with the exact car collision timestamp (time point 0).

Figure 3 illustrates the five overlapping sliding windows across all car collision trials. Specifically, “SW #1" through “SW #5" designate sliding windows spanning from [–2, –1] s to [0, 1] s, with each adjacent window overlapping by 50%. To balance temporal resolution and model estimation stability, five overlapping 1-s sliding windows (50% overlap) were applied across the following intervals: [–2, –1], [–1.5, –0.5], [–1, 0], [–0.5, +0.5], and [0, +1]. At a 500 Hz sampling rate, each window contained 500 data points, sufficient for the specified model order (*p* = 12). The overlapping design ensured temporal continuity and improved sensitivity to transient changes in brain connectivity.

**Figure 4:**
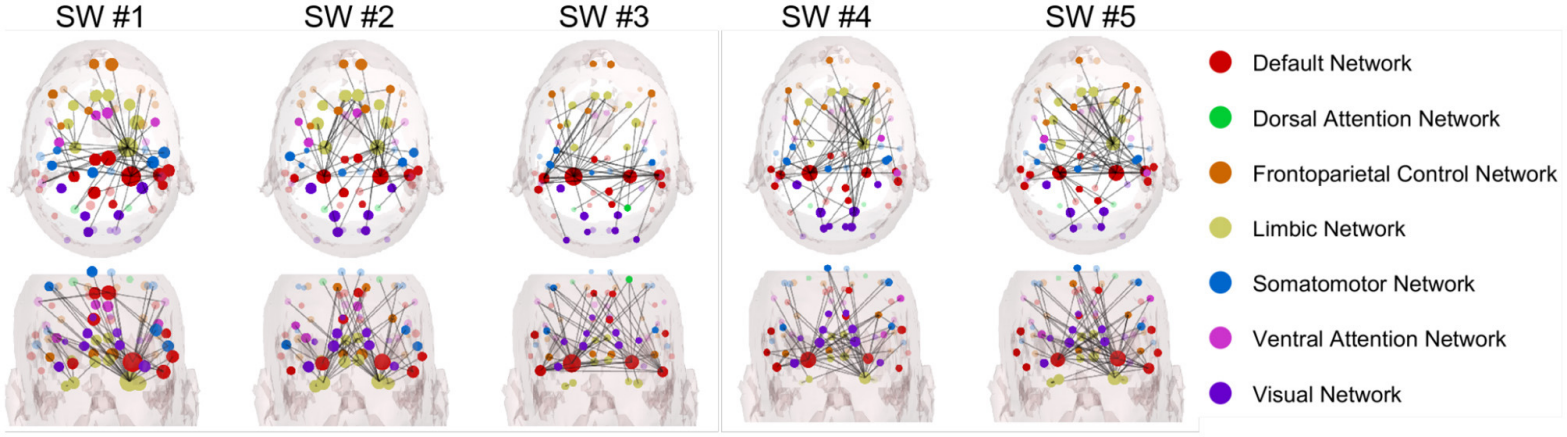
Node strength networks based on Granger causality in the alpha band during car collision trials for the experienced driver group.

**Figure 5:**
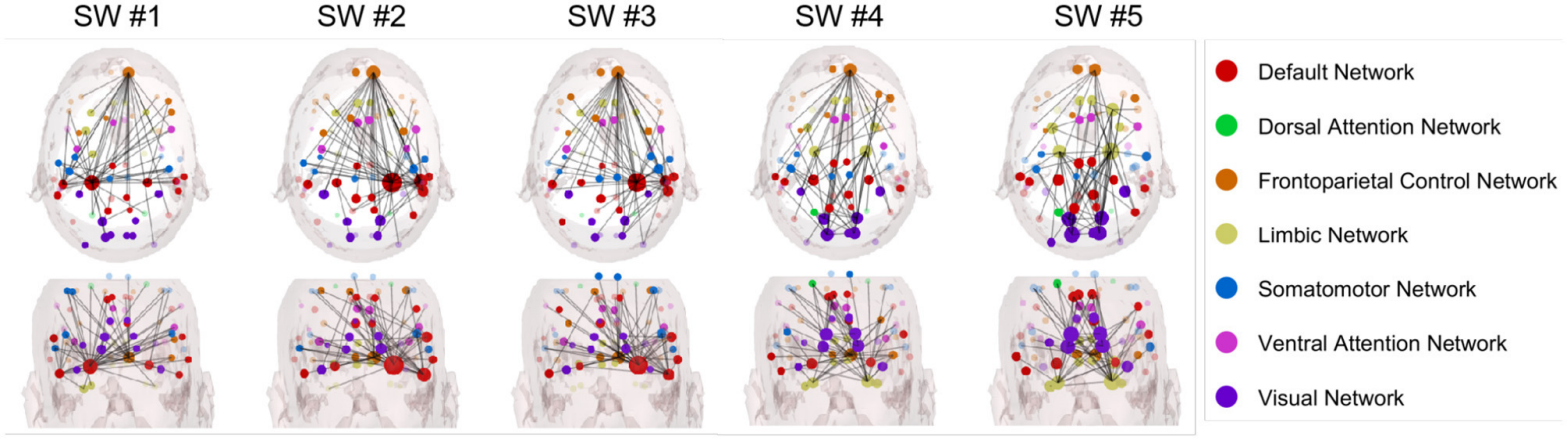
Node strength networks based on Granger causality in the alpha band during car collision trials for the novice driver group.

Figures 4, 5 present the summed in-strength and out-strength Granger causality metrics during car collision trials (with a 50% occurrence rate) for two groups: novice and experienced drivers in car collision risk situations. Granger causality is used to trace and analyze the car collision trials induced time-series EEG epochs, and determine whether one EEG signal can help predict another.

Specifically, Figure 4 presents the dynamic connectivity changes anchored to the car collision time point (set as time zero) for the group of experienced drivers in the alpha band. In contrast, Figure 5 illustrates the corresponding network for novice drivers, providing a comparative perspective. Specifically, for experienced driver group in the alpha band, the summed in-strength and out-strength Granger causality network exhibits the following characteristics:

- In SW #1, experienced drivers show noticeable connections between the visual network (blue) and the default mode network (red), but these connections appear more distributed. From the coronal view, the connectivity in the right temporal lobe is higher and more dense than the left.
- In SW #2, connectivity between the visual network and other networks becomes increasingly complex; however, the connections involving the dorsal attention network (green) and the FCN (yellow) do not show a significant increase. Most connections still primarily involve extensive interactions between the visual network and other networks.
- In SW #3, network interactions become more complex, with connections increasingly distributed across multiple networks, particularly between the default network and the visual network. Connectivity within both temporal regions begins to strengthen, becoming more dominant.
- In SW #4, mean connection strength increases slightly but remains lower than that observed in the novice group, and the lateral connectivity between left and right temporal lobe remains robust, while whole-brain connectivity appears more evenly distributed. Car collision time point is in the center of this SW, visual network is more connected than adjacent SWs. However, in contrast to novice drivers, the connection is relatively sparse.
- In SW #5, connectivity remains relatively stable compared to SW #4.

As for novice drivers group in the alpha band in Figure 5, Granger causality network has prominently connectivity features, as listed below:

- In SW #1, the connectivity between the visual network (blue) and the default mode network (red) are notably prominent.
- In SW #2, the connectivity between visual network and other networks becomes increasingly complex; however, the connections involving the dorsal attention network (green) and FCN (yellow) do not show a significant increase. Most connections still primarily involve extensive interactions between visual network and other networks.
- In SW #3, network interactions become more intricate, with an increased number of connections among multiple networks, particularly between the default network and the visual network.
- In SW #4, the comprehensive connection density continues to rise, with more frequent interactions occurring among different networks.
- In SW #5, the connectivity reaches its peak over all SWs, with highly complex interactions among multiple networks. Visual network connectivity continues to decrease, which forms a striking contrast with the dense connectivity observed in the visual network of the novice driver group.

Broadly speaking, novice drivers exhibit more frequent and complex interactions among brain networks when confronted with danger, whereas experienced drivers demonstrate more stable and dispersed connectivity. This pattern may indicate that experienced drivers are able to process risk situations more efficiently by leveraging their prior experience, thereby reducing the need for extensive network interactions observed in novices.

**Figure 6:**
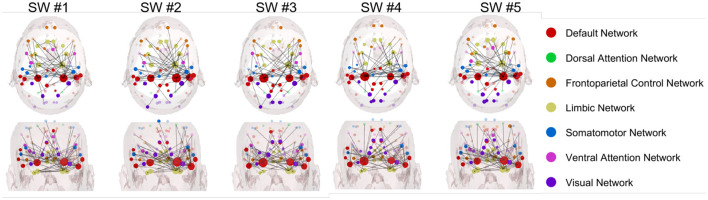
Node strength networks based on Granger causality in the beta band during car collision trials for the experienced driver group.

**Figure 7:**
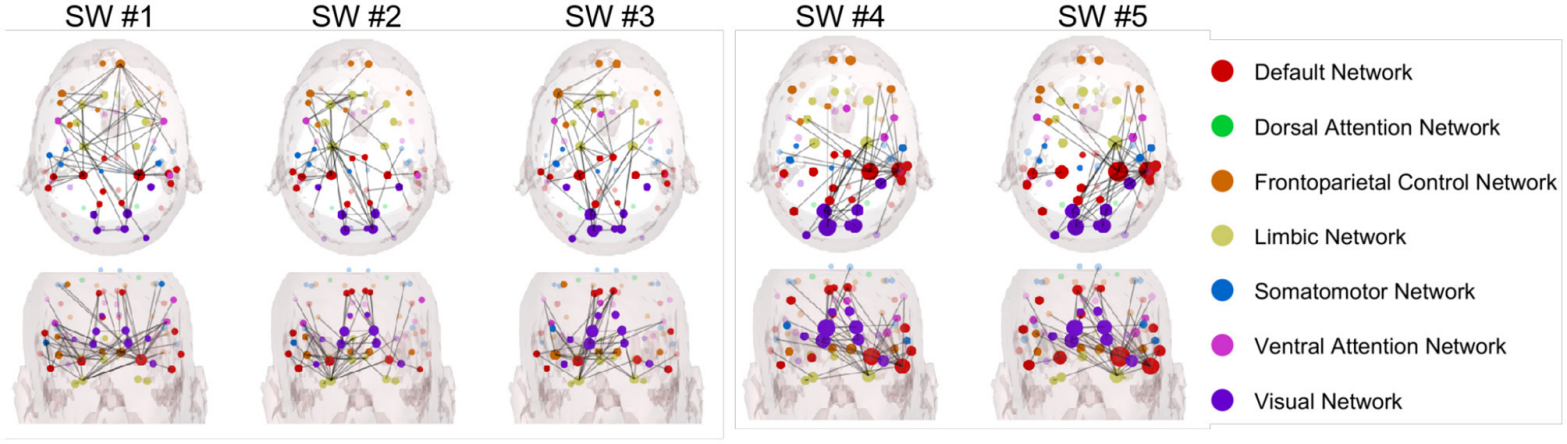
Node strength networks based on Granger causality in the beta band during car collision trials for the novice driver group.

Figures 6, 7 illustrate the Granger-causality-based node strength connectivity networks across five sliding windows with axial and coronal views in the beta band. In Figure 6, the dynamic connectivity variation of the experienced driver group can be summarized below:

- In SW #1, the axial view reveals dense network connections, particularly between the DMN (red) and the visual network (blue). The coronal view displays a relatively uniform distribution of these connections. The strong interhemispheric connectivity between the left and right temporal lobes observed in experienced drivers during the viewing of car collision videos suggests enhanced coordination in information processing and emotional regulation. This finding aligns with studies indicating that experienced drivers exhibit more efficient integration of information and reduced occurrence of "looked-but-failed-to-see" errors when confronted with critical road events.
- In SW #2, a more intricate and denser network of connections, with a greater number of linkages observed between DMN and other networks. Meanwhile, the interhemispheric connectivity between the left and right temporal lobes is comparably enhanced.
- In SW #3, compared to adjacent SWs, the connectivity remains relatively stable, and the interhemispheric connections between the left and right hemispheres have become more pronounced. Among all SWs, SW #3 is the highest level of NS connections over all SWs, with extremely dense interactions observed, particularly between DMN and visual network.
- In SW #4, the axial view reveals a slight decrease in network connection density; however, the connectivity between the left and right temporal lobes remains highly concentrated and relatively stable. The coronal view shows a consistently uniform and strong distribution of connections.
- In SW #5, the density of network connections slightly decreases but remains relatively high.

In Figure 7, the dynamic changes observed in novice drivers in the beta band can be outlined as follows:

- In SW #1, the axial view reveals relatively sparse network connections with weak connection strength comparing to experienced drivers' group, and the frontal eye fields of DAN is intensively connected, which are collectively involved in spatial attention, target localization, and visuomotor control.
- In SW #2, although the axial view displays more connections compared to SW #1, the overall density and strength remain lower than experienced driver group. The dynamic functional connection variation from SW #1 to SW #2 seems less stable and consist for novice group. The coronal view also shows a slight increase in connection density and strength, but it still lags behind experienced drivers.
- In SW #3, the axial view shows a steady increase in connection strength, yet it remain significantly lower than those observed in experienced drivers. Consequently, the overall network complexity and global efficiency are still limited. Meanwhile, the coronal view reveals a noticeably denser connectivity pattern in the left hemisphere compared to the right.
- In SW #4, the connection density continues to approach that of experienced drivers but still exhibits noticeable differences. Notably, within the visual network of the occipital lobe, the connections are denser than those observed in experienced drivers. Considering that SW #4 spans the time window *t*_*epoch*_∈[−0.5, 0.5], centered around the car collision at time zero, it can be inferred that novice drivers focus more on the collision's outcome, engaging in self-referential thinking and heightened sensory processing. In contrast, experienced drivers exhibit a more balanced activation pattern, with greater engagement of regions associated with visual attention and decision-making, leading to more efficient car collision perception.
- In SW #5, node connections continue to approach those of experienced drivers, but differences remain. The connections between right temporal lobe and visual network is prominent, and this integration may indicate that novice drivers, when confronted with collision scenes, show a more intense emotional response and self-referential processing.

### 3.3 Statistical analysis results

#### 3.3.1 Global network metrics analysis

As for the global network metrics analysis, the between-group comparisons on global network metrics revealed consistent patterns of differences between experienced drivers and novices across various frequency bands, conditions, and time-variant sliding windows.

The network-level analysis revealed distinct patterns of connectivity differences between experienced and novice drivers across major brain networks.

#### 3.3.2 Alpha band networks

In the alpha band, we observed generally no significant differences between experienced and novice drivers across most brain networks. Limited significant differences emerged in only a few specific instances: the FCN in SW 5 of the collision condition [*t*_(303)_ = −2.441, *p*_*FDR*_ = 0.036, Cohen's *d* = –0.280], and the DAN in SW 3 [*t*_(308)_ = −2.321, *p*_*FDR*_ = 0.047, Cohen's *d* = –0.264] and SW 5 [*t*_(292)_ = −2.344, *p*_*FDR*_ = 0.045, Cohen's *d* = –0.275)] of the non-collision condition.

#### 3.3.3 Beta band networks

In the beta band, the Granger causality based node strength network differences were substantial and widespread. In car collision condition, all seven major brain networks showed significant differences in SW 1–4, with most differences persisting in SW 5:

- Default mode network: significant differences across all five SWs (all *p*_*FDR*_ < 0.001, Cohen's *d* < –0.585)
- Dorsal attention network: significant in SW 1–4 (all *p*_*FDR*_ < 0.01, Cohen's *d* < –0.380)
- Frontoparietal control network: significant across all five SWs (all *p*_*FDR*_ < 0.05, Cohen's *d* < –0.380)
- Limbic network: significant across all five SWs (all *p*_*FDR*_ < 0.001, Cohen's *d* < –0.496)
- Somatomotor network: significant across all five SWs (all *p*_*FDR*_ < 0.01, Cohen's *d* < –0.432)
- Ventral attention network: significant across all five SWs (all *p*_*FDR*_ < 0.001, Cohen's *d* < –0.479)
- Visual network: significant across all five SWs (all *p*_*FDR*_ < 0.001, Cohen's *d* < –0.698)

In the non-collision condition, five networks showed consistent differences:

- Default mode network: significant across all five SWs (all *p*_*FDR*_ < 0.05, Cohen's *d* < –0.280)
- Frontoparietal control network: significant across all five SWs (all *p*_*FDR*_ < 0.001, Cohen's *d* < –0.421)
- Limbic network: significant across all five SWs (all *p*_*FDR*_ < 0.001, Cohen's *d* < –0.420)
- Ventral attention network: significant across all five SWs (all *p*_*FDR*_ < 0.05, Cohen's *d* < –0.299)
- Visual network: significant across all five SWs (all *p*_*FDR*_ < 0.001, Cohen's *d* < –0.442)

Furthermore, the characteristic path length and global efficiency metrics are also employed to comprehensively analyze the statistical results. Specifically, In the beta frequency band, significant group differences emerged for characteristic path length in both experimental conditions. For the collision condition, novice drivers exhibited significantly higher characteristic path length values compared to experienced drivers across all sliding windows: SW #1 [*t*_(268)_ = −4.219, *p*_*FDR*_ < 0.001], SW #2 [*t*_(276)_ = −4.499, *p*_*FDR*_ < 0.001], SW #3 [*t*_(296)_ = −4.307, *p*_*FDR*_ < 0.001], SW #4 [*t*_(320)_ = −4.814, *p*_*FDR*_ < 0.001], and SW #5 [*t*_(337)_ = −3.560, *p*_*FDR*_ = 0.002]. This pattern was similarly observed in the no-collision condition across all windows: SW #1 [*t*_(317)_ = −3.485, *p*_*FDR*_ = 0.002], SW #2 [*t*_(285)_ = −3.750, *p*_*FDR*_ = 0.001], SW #3 [*t*_(340)_ = −2.987, *p*_*FDR*_ = 0.009],SW #4 [*t*_(352)_ = −3.123, *p*_*FDR*_ = 0.006], and SW #5 [*t*_(342)_ = −3.598, *p*_*FDR*_ = 0.002]. The consistently lower characteristic path length in experienced drivers suggests more efficient information transfer across brain regions.

Global efficiency, which quantifies the network's capacity for parallel information processing, showed pronounced group differences in the beta band for both experimental conditions. For collision scenarios, novices demonstrated significantly lower global efficiency than experienced drivers in all sliding windows: SW #1 [*t*_(187)_ = −4.848, *p*_*FDR*_ < 0.001], SW #2 [*t*_(191)_ = −5.405, *p*_*FDR*_ < 0.001], SW #3 [*t*_(206)_ = −5.928, *p*_*FDR*_ < 0.001], SW #4 [*t*_(238)_ = −6.094, *p*_*FDR*_ < 0.001], and SW #5 [*t*_(255)_ = −4.708, *p*_*FDR*_ < 0.001]. Similarly, in no-collision scenarios, the effect persisted across all windows: SW #1 [*t*_(304)_ = −3.475, *p*_*FDR*_ = 0.002], SW #2 [*t*_(294)_ = −3.732, *p*_*FDR*_ = 0.001], SW #3 [*t*_(308)_ = −3.406, *p*_*FDR*_ = 0.003], SW #4 [*t*_(346)_ = −3.283, *p*_*FDR*_ = 0.004], and SW #5 [*t*_(316)_ = −4.037, *p*_*FDR*_ < 0.001]. These findings indicate that experienced drivers maintain higher information integration capacity throughout the entire collision prediction task.

Across all networks and conditions in the beta band, novice drivers consistently demonstrated higher connectivity values compared to experienced drivers, suggesting fundamental differences in neural processing strategies during driving-related decision-making. In addition, the experimental results revealed no significant differences between groups in either reaction time or accuracy.

## 4 Discussion

In this study, we conduct a comprehensive analysis of EEG-based dynamic brain networks using Granger causality, with node strength employed to examine the differences between two groups. The following sections provide a detailed discussion of the whole brain and intrinsic functional network-based analysis.

### 4.1 Comprehensive discussions on dynamics of Granger causality-based brain network

As for the alpha band, the between-group EEG differences suggest that these observation that experienced drivers maintain more stable and distributed network connectivity patterns, potentially reflecting efficient neural strategies for integrating sensory information and decision-making during driving tasks. In contrast, novice drivers exhibit progressively increasing connectivity complexity, which may indicate a less efficient or more effortful neural processing strategy as they respond to driving-related stimuli.

Since the beta band EEG is closely associated with various brain functions, including alertness, risk avoidance, attention allocation, and motor intuition. It also has potential impacts on cognitive control, working memory, motor coordination, motor skill learning, emotional arousal, and stress responses. In the following, we focus on the beta band and provide further analysis and interpretation of the connectivity network results in Figure 4.

In our work, specifically in the beta band, the observed differences between experienced and novice drivers offer further insights into how brain networks function differently in each group. The beta band is commonly associated with higher cognitive functions, such as motor control, attention, and cognitive processing, making it a critical band for investigating the neural mechanisms underlying first-person car collision risk prediction performance. In the following, we summarize a more detailed analysis of the EEG differences between two groups, as listed below.

- Connectivity and synchronization. The beta band in experienced drivers shows highly synchronized network activity, particularly between DMN and the visual network. It suggests more efficient communication between regions responsible for self-referential thinking and sensory processing. The greater synchronization observed in the beta band may reflect better coordination of cognitive processes, such as attention and visual processing, which are crucial for quick decision-making and effective driving. In contrast, novice drivers exhibit less synchronized. The reduced connectivity, especially between networks like the DMN and the visual network, could indicate slower cognitive processing and less efficient integration of sensory and cognitive information. This is consistent with their increased susceptibility to distractions and delayed decision-making during driving, as their brain networks are not as optimized for such complex tasks.
- Cognitive control. The increased beta band power in the group of experienced drivers, particularly in the axial view, suggests that their brains are more engaged in maintaining cognitive control. The heightened beta band power could indicate that experienced drivers are better at suppressing irrelevant stimuli and focusing their attention on driving tasks, which is crucial for safe and efficient driving. Compared to this, beta power in novice drivers, may be lower, which could reflect weaker cognitive control mechanisms. A lower beta band power might suggest that novice drivers struggle more with filtering out distractions and maintaining sustained attention on the driving task. This decreased beta activity could be linked to their higher level of cognitive load and greater variability in task performance, especially under complex or challenging driving conditions.
- Network complexity and adaptability. A more complex network connectivity observed in experienced drivers across all SWs, indicates a higher level of adaptability and flexibility in brain functioning. The Granger causality-based connectivity networks are not only denser but also more capable of dynamically switching between different cognitive networks. This flexibility likely reflects their ability to adjust quickly to varying car driving conditions and make decisions with a high degree of confidence and precision. In contrast, the networks of novice drivers remain relatively simpler and less adaptable, particularly in the earlier time windows, which suggests they are not capable of rapidly switching between different brain regions and respond efficiently to driving scenario. This could explain why novice drivers may struggle more with unexpected situations and require more time to react to stimuli, as their brain networks are still developing the necessary complexity and adaptability for optimal performance.
- Stability and consistency. The stability of beta band activity in experienced drivers, especially in the later windows (SW #4 and SW #5), suggests that they have developed a reliable and consistent neural framework for handling complex driving experience tasks. Their ability to maintain relatively stable network activity over time may reflect a well-established neural pathway for managing cognitive demands during driving. This stability is essential for reducing errors and maintaining safe driving behavior under varying conditions. Compared to that, greater variability in network connectivity seen in novice drivers, especially in the earlier windows, indicates that their brain networks are less stable and more prone to fluctuations. This instability in beta band activity could be linked to the greater cognitive load they experience while driving. As novice drivers are still learning and refining their driving skills, their brains may have to work harder to process information and make decisions, leading to greater fluctuations in their neural activity.
- Implications for training and expertise development. The differences between the two groups suggest that brain training could be a valuable tool for assessing driving performance, as it not only enhances driving skills but also serves as a scientific, objective method by measuring cognitive abilities, detecting skill differences, optimizing training, and providing reliable evaluation criteria for both novice drivers and examiners.

### 4.2 Discussions on the dynamic functional network

**Figure 8:**
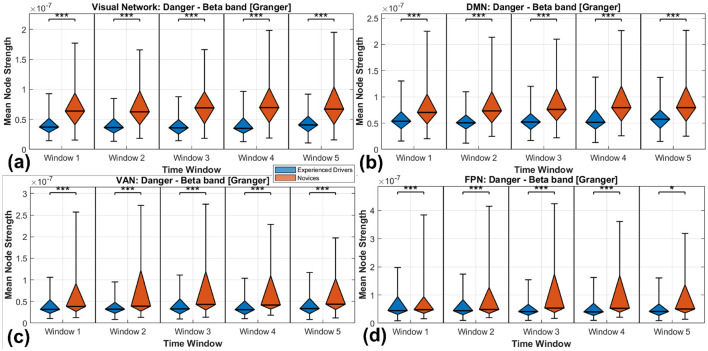
Distribution of mean NS across four functional networks: **(a)** visual network; **(b)** DMN; **(c)** VAN; **(d)** FCN. *p < 0.05, ***p < 0.001.

In this subsection, the dynamic intrinsic functional network based node strength over experienced drivers and novices during five overlapped sliding windows are investigated. Specifically, the visual network, DMN, VAN and FCN are extensively discusses, as shown in Figure 8. The following is a detailed analysis and comparison of each sub-figure.

- Visual network. Novice drivers show significantly higher node strength than experienced drivers, indicating that novice drivers exhibit stronger or more frequent responses to danger in the visual network.
- DMN. As for experienced drivers, node strength remains relatively stable across all time windows, slightly lower than that of novice drivers.
- VAN. Novice drivers show significantly higher node strength than experienced drivers, indicating that novice drivers have stronger or more frequent responses to danger in the ventral attention network.
- FCN. As time progresses, the mean node strength for experienced drivers gradually decreases. In contrast, novice drivers exhibit an initial increase in mean node strength followed by a decrease, accompanied by a relatively weaker prediction of ongoing car collision events.

From the perspective of a car collision risk study, Figures 8a–8d suggest:

- Stronger responses in novice drivers. In all four networks, novice drivers show significantly higher NS than experienced drivers, indicating that their brain networks respond more strongly or more frequently to danger.
- Greater stability in experienced drivers. Experienced drivers maintained relatively stable and lower NS across all networks, which may indicate that they have learned to better control and regulate their responses to danger, thereby reducing unnecessary neural activity.
- Network differences. Although the overall trends in the four intrinsic functional networks are similar, the specific response patterns in each network may reflect the distinct roles of different brain regions in processing danger-related information. For instance, the visual network and DMN may exhibit more prominent responses during initial perception and in a resting state, whereas the ventral attention and prefrontal control networks may play a more significant role in attention allocation and control.
- Superior risk perception abilities. Experienced drivers exhibit superior hazard perception abilities compared to novice drivers. This difference is reflected in their brain activity, with experienced drivers showing increased activation in regions associated with visual attention and decision-making. They also demonstrate higher functional connectivity between brain areas responsible for processing visual information and those involved in assessing the salience of potential hazards. These neural enhancements contribute to their improved capacity to anticipate and respond to potential car collision events.

In summary, the beta band EEG differences between experienced and novice drivers reflect significant differences in brain network coordination, cognitive control, adaptability, and stability. Experienced drivers show stronger, more complex, and more stable beta band activity, which facilitates better decision-making, attention regulation, and motor control. Novice drivers, on the other hand, exhibit less synchronized, weaker, and less stable beta band activity, which could contribute to the cognitive challenges they face while driving. These findings highlight the potential of EEG-based biomarkers in understanding the neural mechanisms of driving expertise and suggest that targeted training could help novice drivers optimize their brain network functioning.

## 5 Conclusion

In this study, we successfully demonstrated the feasibility of using EEG-based Granger causality analysis to predict collision risk scenarios. Experienced drivers exhibited more stable and distributed network connectivity patterns compared to novice drivers, indicating more efficient neural strategies for integrating sensory information and decision-making during driving tasks. The beta band EEG activity in experienced drivers showed higher synchronization, particularly between DMN and the visual network, reflecting better coordination of cognitive processes crucial for quick decision-making and effective driving. In particular, we identified higher synchronization in the beta band between DMN and visual network, which can be interpreted as evidence for better coordination of cognitive processes during decision making. Through this analysis, we have demonstrated that naturalistic training alone is effective at changing functional circuits. This allows future research to identify different biomarkers for action/decision making detection dependent on experience level. These findings and methodologies presented here lay a solid foundation for future research aimed at improving driving safety through enhanced understanding and training of intuitive driving skills.

## Data Availability

The raw data supporting the conclusions of this article will be made available by the authors, without undue reservation.
